# The local translation of *K_Na_* in dendritic projections of auditory neurons and the roles of *K_Na_* in the transition from hidden to overt hearing loss

**DOI:** 10.18632/aging.102553

**Published:** 2019-12-08

**Authors:** Jeong Han Lee, Mincheol Kang, Seojin Park, Maria C. Perez-Flores, Xiao-Dong Zhang, Wenying Wang, Michael Anne Gratton, Nipavan Chiamvimonvat, Ebenezer N. Yamoah

**Affiliations:** 1 Department of Physiology and Cell Biology, School of Medicine, University of Nevada Reno, Reno, NV 89557, USA; 2 Department of Internal Medicine, Division of Cardiology, University of California Davis, Davis, CA 95616, USA; 3 Department of Otolaryngology, Head and Neck Surgery, Washington University St. Louis, St. Louis, MO 63110, USA

**Keywords:** hearing loss, potassium channels, age-related hearing loss, auditory neurons, axonal protein translation

## Abstract

Local and privileged expression of dendritic proteins allows segregation of distinct functions in a single neuron but may represent one of the underlying mechanisms for early and insidious presentation of sensory neuropathy. Tangible characteristics of early hearing loss (HL) are defined in correlation with nascent hidden hearing loss (HHL) in humans and animal models. Despite the plethora of causes of HL, only two prevailing mechanisms for HHL have been identified, and in both cases, common structural deficits are implicated in inner hair cell synapses, and demyelination of the auditory nerve (AN). We uncovered that Na^+^-activated K^+^ (K_Na_) mRNA and channel proteins are distinctly and locally expressed in dendritic projections of primary ANs and genetic deletion of K_Na_ channels (*Kcnt1* and *Kcnt2*) results in the loss of proper AN synaptic function, characterized as HHL, without structural synaptic alterations. We further demonstrate that the local functional synaptic alterations transition from HHL to increased hearing-threshold, which entails changes in global Ca^2+^ homeostasis, activation of caspases 3/9, impaired regulation of inositol triphosphate receptor 1 (IP_3_R1), and apoptosis-mediated neurodegeneration. Thus, the present study demonstrates how local synaptic dysfunction results in an apparent latent pathological phenotype (HHL) and, if undetected, can lead to overt HL. It also highlights, for the first time, that HHL can precede structural synaptic dysfunction and AN demyelination. The stepwise cellular mechanisms from HHL to canonical HL are revealed, providing a platform for intervention to prevent lasting and irreversible age-related hearing loss (ARHL).

## INTRODUCTION

Previous studies have demonstrated distinct local domain-specific protein translation as a mechanism to confer multiple temporal and spatial neuronal functions [1]. However, the merits of distinct functional segregations may be offset by local and latent neuropathology that can escape early detection, an overture for intervention. Auditory information processing relies heavily on precise temporal signal transmission [2]. Loss of sound segregation [3] is one of the emergent, early signs of age-related hearing loss (ARHL) [4, 5]. Previously, ARHL was determined by increased audiometric thresholds [6, 7]. Recent studies in humans and animal models have demonstrated that traces of ARHL may begin much earlier than previously predicted. A standard threshold characterizes the early signs of ARHL, but there is a reduced suprathreshold amplitude of sound-elicited compound action potentials (AP), a reduction in peak I, and an increase in latency of the auditory brainstem response (ABR) waveform [8, 9]. Because of the apparent normal threshold but reduced physiological characteristics, the phenomenon has been named hidden hearing loss (HHL) [3, 8, 10, 11]. HHL may degrade spike reliability and precision, and result in compensatory hyperactivation of auditory neurons (AN) in higher brain centers, causing tinnitus [11, 12]. Thus, the prevailing notion is that HHL begets additional auditory deficits and overt increases in audiometric threshold. These findings suggest that early but latent mechanisms underlying the development of ARHL if identified can be repressed, or reversed to prevent progression to overt HL. What are the mechanisms of HHL and the transition steps to overt HL? To date, structural dysfunction of the inner hair cell (IHC) synapse and transient primary AN demyelination have been demonstrated as two of the mechanisms of HHL [3, 8].

The cochlea houses inner hair cells (IHCs) that encode sound information by transducing mechanical stimuli into neurotransmitter release, and electrical signals that are relayed by type I spiral ganglion neurons (SGNs) to the cochlear nucleus (CN) and ascending auditory pathways [13]. Type I SGNs are bipolar neurons that extend unbranched myelinated projections, which form single ribbon synapses with one IHC [14]. A single IHC is innervated by ~6-20 type I SGNs in the mature mouse cochlea. Presynaptic ribbons and postsynaptic densities are marked by C-terminal Binding Protein 2 (CtBP2) [15], a postsynaptic density 95 protein (PSD95), and AMPA-type glutamate receptors [16, 17]. The heminodes of type I afferents are decorated with Na^+^ channels to initiate the rising phase of action potentials (APs) to ensure swift acoustic conduction [18]. Paradoxically, increased Na^+^ current density can produce enhanced membrane instabilities, which disrupt spike synchrony and impede AP conduction speed [19, 20]. The interplay between Na^+^ currents and activation of K^+^ currents may be essential in regulating membrane potential jitters. Indeed, we have recently demonstrated that primary AN expressed Na^+^-activated K^+^ channels, K_Na_1.1 (SLO2.2/Slack), and K_Na_1.2 (SLO2.1/Slick) (K_Na_) [21]. Null deletion of the two genes *Kcnt1* and *Kcnt2* [22], result in HHL [21]. We hypothesized that there are stepwise cellular mechanisms from HHL to overt HL in the *kcnt1* and *kcnt2* null mouse model.

Here, we show that K_Na_ channels mRNA is distinctly and locally expressed in dendritic projections of primary ANs, raising the possibility that the channels play unique roles in synaptic functions. We demonstrate that Na^+^-activated K^+^ channels regulate spike jitters introduced by Na^+^ currents. Null deletion of *Kcnt1* and *Kcnt2*, result in HHL in the absence of structural changes in synaptic morphology. By a persistent Na^+^ current, K_Na_ current contributes toward the resting membrane potential (RMP) of SGNs. Thus, attenuation of K_Na_ current in *Kcnt1* and *Kcnt2* (double knock out (DKO)) in SGNs leads to depolarized RMP, resulting in reduced AP amplitude, translating into ABR peak I amplitude reduction and increased delay, but normal ABR thresholds and synaptic morphology. Owing to local attenuation of K_Na_ current activity, there is a long-term global increase in membrane activity, leading to enhanced intracellular Ca^2+^ (Ca^2+^_i_) and altered Ca^2+^ handling. These changes culminated with a gradual activation of caspase 3/9, impaired regulation of inositol triphosphate receptor 1 (IP_3_R1), and apoptosis-mediated synaptic and neuro-degeneration. The findings demonstrate how a change in local neuronal activity can lead to progressive disease. It also identifies a potential interventional platform to treat ARHL.

## RESULTS

### Local *Kcnt1* and *Kcnt2* mRNA and proteins at postsynaptic terminals and soma of SGNs

**Figure 1:**
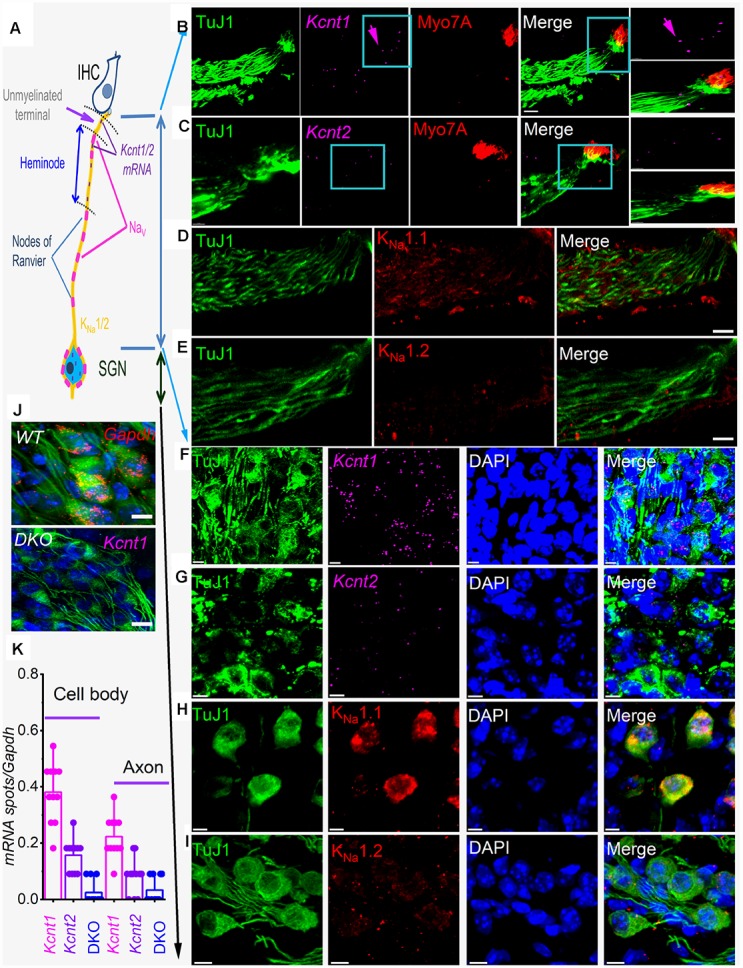
**sm-FISH and immunocytochemistry localize transcripts and proteins for K_Na_1.1 and K_Na_1.2 in axons and cell bodies of spiral ganglion neurons (SGNs).** Expression of K_Na_1-encoding transcripts in the SGNs was examined using smFISH and standard immunocytochemistry in the organ of Corti (OC)/SGN preparations from 1-mo old C57 mice (**B**–**I**). (**A**) Schematic illustration of the inner hair cell (IHC), type I SGN, the peripheral axon, and cell body. The unmyelinated terminal, heminode, and nodes of Ranvier are noted, but not to scale. (**B**) RNA molecules encoding for K_Na_1.1 (*Kcnt1*), and (**C**) K_Na_1.2 (*Kcnt2*), were detected as fluorescent spots (purple, arrow) in TUJ1-positive (green) SGN axons, IHCs were labeled with myosin 7A antibody (red), and merged images are shown. Axonal *Kcnt1* mRNA were prominent, but only scant *Kcnt2* mRNA spots were detected compared to the double knockout (DKO) samples (**J**). Scale bar = 10 μm (**D**–**E**) Images of cochlear sections of 1-mo old mice show that K_Na_1.1 (red) protein is expressed in the auditory nerve in D. Consistent with the faint expression of *Kcnt2* mRNA in the axons in (**E**) there was virtually little or no detectable expression of K_Na_1.2 in axons of the auditory nerve. Scale bar = 10 μm. (**F**–**G**) mRNA spots (purple spots) encoding K_Na_1.1 (*Kcnt1*), and K_Na_1.2 (*Kcnt2*) in the cell bodies of SGNs. Very few spots for *Kcnt2* mRNA were detected. Sections were co-labeled with neuronal (TuJ1, green) and nuclei markers (4,6-diamidino-2-phenylindole, DAPI, blue) Scale bar = 5 μm. (**H**–**I**) Images of the SGNs show K_Na_1.1 (red) protein is expressed in cell bodies of the auditory nerve. In keeping with low levels of expression of mRNA, K_Na_1.2 protein expression was faintly positive. The mean number of RNA molecules detected per SGN was calculated as described in the Methods. *Kcnt1* levels were higher compared to *Kcnt2* in both mRNA and protein levels. (**J**) (Upper panel). Photomicrograph showing SGN mRNA spots (red spots) encoding *Gapdh* (data was obtained from DKO tissue)*.* (Lower panel) DKO cochlear section, using *kcnt1* probe serving as negative controls. Similar data were obtained using the *kcnt2* probe (data not shown). Scale bar = 5 μm. (**K**) Values of mRNA spots in axons and cell bodies were normalized against *Gapdh* mRNA spots/100 μm^2^ (11 ± 2 spots (n = 31)) are summarized in the form of bar graphs. The mean (mean ± SD) was (cell body, *kcnt1* = 0.38 ±.0.11; *kcnt2* = 0.16 ± 0.06; DKO = 0.02 ± 0.04; n = 11 animals; derived from 50 randomly selected cells and evaluated by 5 blinded individuals. The mean (mean ± SD) was (axons, *kcnt1* = 0.22 ±.0.07; *kcnt2* = 0.08 ± 0.06; DKO = 0.03 ± 0.05; n = 11 animals; derived from 50 randomly selected cells and evaluated by 5 blinded individuals.

K_Na_1.1 and K_Na_1.2 have been localized in the medial nucleus of the trapezoid body (MNTB) in the auditory brainstem and shown to regulate spike timing [24, 25] and in peripheral neurons in the dorsal root ganglion (DRG), where they regulate nociceptive responses [23, 26, 27]. However, *Kcnt1* and *Kcnt2* mRNA have recently been localized in SGN cell bodies [21]. To determine the roles of the K_Na_1 channels, we examined the expression pattern of mRNA and protein in SGNs. Figure 1A provides a schematic diagram of an IHC and SGN for orientation. In addition to the expected localization of mRNA in SGNs soma, where the channels are synthesized, *Kcnt1,* and *Kcnt2* mRNA were surprisingly detected at the synaptic projections (Figure 1B–1E). The expression levels of *Kcnt1* were consistently higher than *Kcnt2* in SGNs (Figure 1). Local protein translation has been identified to be essential for axonal maintenance [28], dendritic functions [29–31], and synaptic plasticity [32]. Indeed, localized axonal K^+^ channel translation has been reported [33]. We examined the expression of K_Na_1.1 and K_Na_1.2 in different compartments of SGNs. K_Na_1.1 was densely, and K_Na_1.2 was faintly expressed at the cell body and dendritic projections (Figure 1D, 1E, 1H, 1I). Local expression of K_Na_ channels suggests that channel activity may regulate synaptic function and axonal action potential (AP) conduction.

Furthermore, the distribution of the channel proteins along the soma-dendrite axis, including the heminodal region, varied, raising the possibility that K_Na_ channels may confer distinct functions. The heminode of the peripheral axon is the spike initiation site with high Na^+^ channel density [34] and the origin of auditory information coding [35]. We reasoned that K_Na_1 regulates Na^+^ channel activity, and the null deletion of K_Na_1 channels may have a functional impact on SGN properties and overall pre-CN auditory function. To address the functional roles of K_Na_1 channels, we took advantage of a DKO mouse model [23].

### Null deletion of *Kcnt1* and *Kcnt2* (DKO) resulted in HHL and gradual transition to overt hearing loss

**Figure 2:**
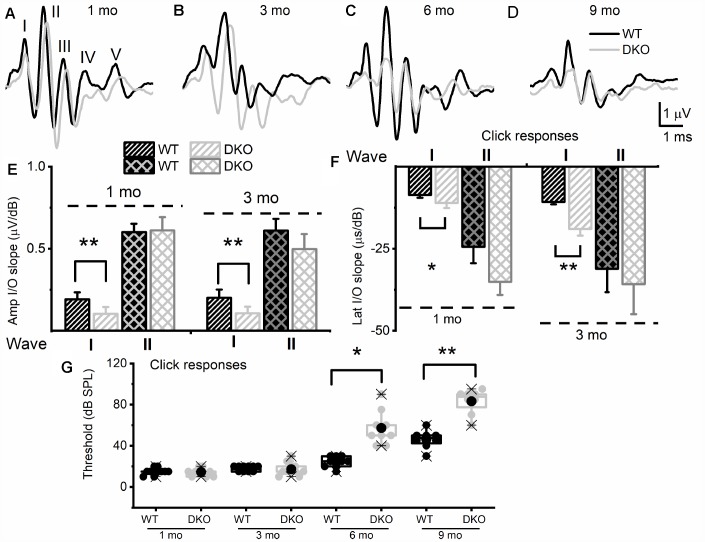
**K_Na_1 DKO mice have reduced and delayed wave I auditory brainstem responses (ABR) but normal absolute thresholds from ages 1 to 3-mo-old in comparison with WT animals, and by 6-mo-old, showed a profound increase in absolute thresholds, relative to the WTs.** ABRs were measured in 1-, 3-, 6- and 9-mo old WT and DKO mice. (**A**–**D**) Representative ABR traces in response to 80 dB SPL sound clicks are shown for WT (black) and DKO (light grey), at ages 1-, 3-, 6- and 9-mo old as indicated. (**E**) Average waves I and II amplitudes (input/output, I/O) linear regression slope functions were determined for responses to sound clicks. Wave I, but not wave II, amplitudes were significantly reduced in DKO mice compared to WT mice in response to click sound in 1-mo old. In 3-mo old wave II was significantly different. The mean values for 1-mo old mice for wave I (I/O slope (μV/dB); mean ± SD) were; WT = 0.19 ±.0.04; DKO = 0.10 ± 0.04; n = 11; *p <* 0.0001. Mean wave II values were; WT = 0.60 ±.0.05; DKO = 0.61 ± 0.08; n = 11; *p* = 0.7. The mean values for 3-mo-old mice for wave I (I/O slope (μV/dB); mean ± SD) were; WT = 0.20 ±.0.05; DKO = 0.11 ± 0.04; n = 11; *p <* 0.0001. Mean wave II values were; WT = 0.61 ±.0.07; DKO = 0.50 ± 0.09; n = 11; *p* = 0.004. (**F**) Average waves I and II latency (input/output, I/O) functions versus sound clicks. Wave I latency I/O linear regression slopes were significantly different between WT and DKO mice. The mean values for 1-mo old mice for wave I latency (I/O slope (μs/dB); mean ± SD) were; WT = 8.60 ±.0.82; DKO = 11.05 ± 1.57; n = 11; *p <* 0.0002. Mean wave II values were; WT = 24.40 ±.5.01; DKO = 35.09 ± 4.02; n = 11; *p* = 0.0001. The mean values for 3-mo old mice for wave I (I/O slope (μV/dB); mean ± SD) were; WT = 10.73 ±.0.73; DKO = 18.93 ± 2.02; n = 11; *p <* 0.0001. Mean wave II values were; WT = 31.09 ±. 7.11; DKO = 35.80 ± 9.12; n = 11; *p* = 0.192. Additionally, the I/O slope (μV/dB) of wave I amplitude at 6-mos and 9-mos were; WT = 0.16 ± 0.05; DKO = 0.08 ± 0.06; n = 10; *p* = 0.005 and (9-mos); WT = 0.14 ± 0.08; DKO = 0.05 ± 0.02; n = 10; *p* = 0.04. The I/O slope (μs/dB) of wave I latency at 6-mos and 9-mos were; WT = 12.37 ± 0.91; DKO = 20.81 ± 3.11; n = 10; *p* < 0.0001 and (9-mos); WT = 13.21 ± 1.06; DKO = 22.63 ± 4.11; n = 10; *p* < 0.0001. (**G**) Mean absolute ABR thresholds in response to click stimulus were not statistically significantly different between WT and DKO mice in 1-3 mo old but were significantly different in 6- and 9-mo old mice. The mean values for ABR thresholds for 6-mo old were (dB SPL); WT = 24.44 ± 5.27; DKO = 57.22 ± 16.41; n = 9; *p* = 0.0002. Mean thresholds for or 9-mo old were; WT 46.25 ± 8.76; DKO = 83.13 ± 11.93; n = 8; *p* < 0.0001.

**Figure 3:**
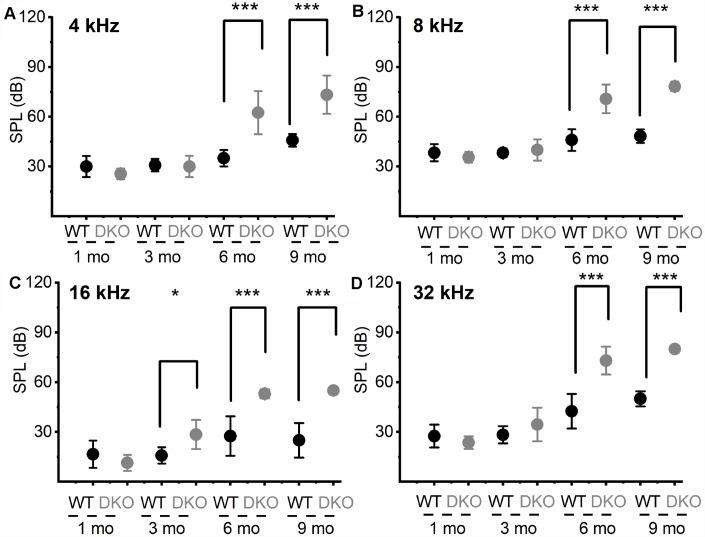
**Changes in pure tone responses.** (**A**–**D**) Mean absolute ABR thresholds in response to pure tones (4, 8, 16 and 32 kHz) were not statistically significantly different between WT and DKO mice in 1- and 3-mo old, but were significantly different in 6- and 9-mo old mice. Summary data are expressed as (mean ± SD), and statistical comparisons are shown. For 4 KHz, the mean thresholds (in dB SPL) at 6 mos were; WT = 35.00 ± 5.00; DKO = 62.50 ± 12.94; n = 8; *p* = 0.0001. Mean thresholds at 9 mos were; WT = 45.83 ± 3.76; DKO = 73.33 ± 11.55; n = 8; *p* = 0.0001. For 8 KHz, the mean thresholds (in dB SPL) at 6 mos were; WT = 46.00 ± 6.52; DKO = 70.83 ± 8.60; n = 8; *p* = 0.0001. Mean thresholds at 9 mos were; WT = 48.30 ± 4.10; DKO = 78.30 ± 2.89; n = 8; *p* = 0.0001. For 16 KHz, the mean thresholds (in dB SPL) at 3 mos were; WT = 15.83 ± 4.91; DKO = 28.50 ± 8.83; n = 8; *p* = 0.0032. Mean thresholds at 6 mos were; WT = 27.50 ± 11.90; DKO = 53.00 ± 2.74; n = 8; *p* = 0.0001. Mean thresholds at 9 mos were; WT = 25.00 ± 10.49; DKO = 55.00 ± 0.00; n = 8; p = 0.0001. For 32 KHz, the mean thresholds (in dB SPL) at 6 mos were; WT = 42.50 ± 10.40; DKO = 73.84 ± 8.37; n = 8; *p* = 0.0001. Mean thresholds at 9 mos were; WT = 50.00 ± 4.47; DKO = 80.00 ± 0.00; n = 8; *p* = 0.0001.

To test the hypothesis that deletion of K_Na_1.1 and K_Na_1.2 might cause short- and long-term auditory deficits, we performed ABRs analyses. We determined not only the sound pressure levels (SPL) at which the characteristic ABR waveform could be detected but also changes in wave (peak) one/two (PI/PII) amplitudes and latencies with respect to sound stimulus intensity. The motivation for the latter was that the origin of PI/PII might stem from type I afferent postsynaptic terminal [36, 37] or at the soma [38] (pre-CN), where K_Na_1.1 and K_Na_1.2 expression is high (Figure 1). The analyses were performed for 1-, 3-, 6- and 9-months (mo) old DKO mutant and their age-matched C57 WT mice. The sound stimuli were broadband click (Figure 2) and pure tones at 4, 8, 16, and 32 kHz (Figure 3). WT mice yielded the characteristic ABR waveform at 1 mo of age with thresholds of 20-30 dB across different test frequencies. The hearing remained unchanged until 6-9-mo old when the threshold increased to 40-45 SPL. For the example shown in Figure 2A–2D, all five ABR peaks were apparent in response to an 80-dB stimulus. In contrast, DKO mice exhibited a markedly reduced PI amplitude waveform compared to the WT at all ages (1-9 mo). Analyses of the input/output (I/O) function of PI and PII amplitude and latency between WT and DKO across ages showed that while PI amplitudes and latencies were markedly reduced and delayed, respectively, in response to a click stimulus, the thresholds in 1- and 3-mo old DKO were equivalent to those of the WT mice (Figure 2E–2G). However, by 6-mo and beyond, the DKO mice exhibited significant changes in PI and PII amplitudes and latencies. Sound thresholds in response to clicks and pure tones stimuli have increased significantly (Figures 2G, 3). For clarity, data for pure tones 4, 8, 16, and 32 kHz are illustrated in Figure 3. The results from the DKO mice at ages 1-3 mo have a striking resemblance to the physiological signatures of HHL, and in 6 mo and older animals, the HHL has transitioned to canonical HL, whereby there is a significant increase in the absolute hearing threshold (Figures 2G, 3). To understand the cellular mechanisms for alterations of ABR waveforms in the DKO, we examined the SGN response properties, using mice with documented HHL and overt HL.

### HHL in DKO results from increased membrane jitters, and altered SGN response properties

**Figure 4:**
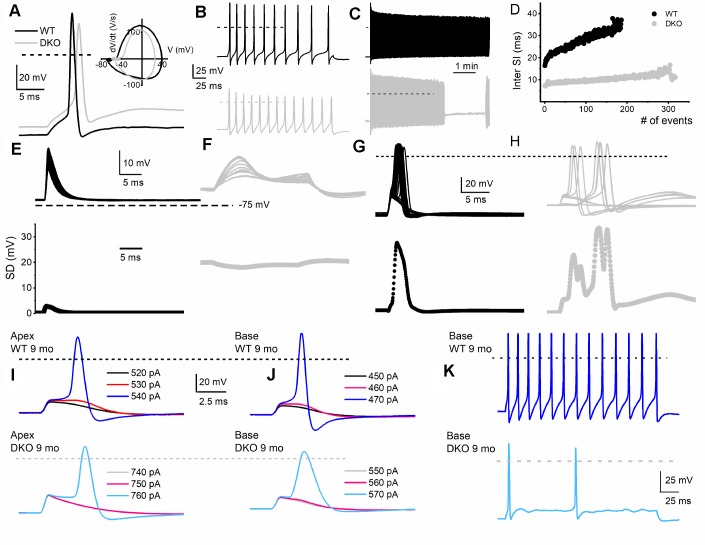
**Membrane properties of SGNs from WT and DKO mice.** To minimize experimental variabilities, SGN recordings were performed using mice, with recorded ABR. Current-clamp recordings were performed on SGNs isolated from the basal and apical one-third of the cochlea from 1-, 3-, 4-5- (not shown), 6- and 9-mo old WT and DKO mice. (**A**) Action potentials (AP) evoked from 1-mo-old SGNs isolated from cochlear apical turn from WT (black trace) and DKO (grey trace) mice. Both the resting membrane potential (RMP) and AP amplitude were significantly altered in WT *versus* DKO SGNs. RMP of apical SGNs; WT, -65 ± 3 mV, n = 31; DKO, -58 ± 2 mV, n = 27; *p* < 0.001: RMP of basal SGNs; WT, -57 ± 2 mV, n = 25; DKO, -53 ± 4 mV, n = 31; *p* < 0.001). AP amplitudes were; apical SGN; WT, 80.0 ± 2.7 mV, n = 15; DKO, 72.1 ± 4.8 mV, n = 15; *p* < 0.001: basal SGN; WT, 83.2 ± 3.4 mV, n = 17; DKO, 72.0 ± 5.1 mV, n = 19; *p* < 0.001). Dotted black and gray lines indicate 0 mV. (**B**, **C**) Examples of slow adapting SGNs isolated from a 1-mo-old basal cochlear turn. For the example shown in (**B**) typical spike frequency (in Hz) for WT SGNs = 45 ± 7 (n = 17) and DKO = 67 ± 11 (n = 21); *p* < 0.0001. (**D**) The dairy plot of the inter-spike interval of slow adapting SGNs from WT and DKO. (**E**, **F**) 50 consecutive subthreshold depolarization (0.075 nA current injection) of WT SGNs (**E**, black traces), and DKO (**F**, grey traces), demonstrating the extent of membrane jitters. Plotted below is the corresponding standard deviation. (**G**, **H**) 30 consecutive suprathreshold depolarization (0.2 nA current injection; interstimulus interval, 2s) of WT SGNs (**G**, black traces), and DKO (**H**, grey traces), demonstrating the extent of membrane jitters in evoked APs. Plotted below is the corresponding standard deviation. DKO membrane voltage is pre-disposed to increased membrane jitters. (**I**, **J**) APs evoked in SGNs from 9-mo old WT (upper panel) cochlea were generally slower to initiate and larger in amplitude compared to those evoked in SGNs from DKO (lower panel) cochlea. The magnitudes of the injected current are indicated. (**K**) Across SGNs, the excitability of DKO SGNs has plummeted by several-fold. For the example shown, the spike frequency was reduced by ~7-fold.

**Figure 5:**
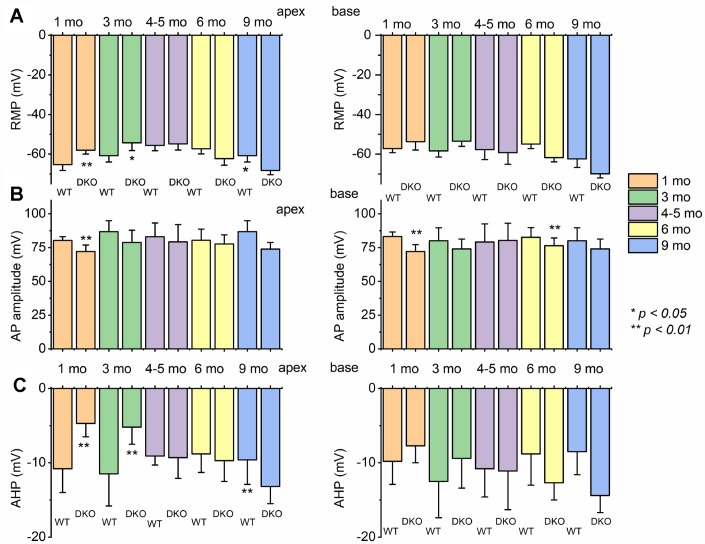
**Changes of membrane properties of WT *versus* DKO SGNs isolated from cochlear apex and base at different ages (1- 3- 4-5- 6- and 9-month).** (**A**) The resting membrane potential (RMP) of SGNs was significantly altered at different stages in development. (**B**) Changes in AP amplitudes of WT and DKO SGNs from apical and basal cochlea are summarized. (**C**) Amplitude of afterhyperpolarization (AHP) measured and summarized (*p < 0.05; **p < 0.01; n = number of SGNs are indicated). For these data, significant differences between genotypes were determined using the unpaired two-tailed *t-test*. Other action potential parameters such as latency and duration were measured but not shown (we did not identify any significant changes in action potential latency and duration in WT versus DKO at different stages of development and location in the cochlea).

To assess the transition from HHL to overt HL in DKO mice requires a longitudinal evaluation of the functional properties of SGNs from 1-9-mo old. Previously, recordings from 1.5-mo old DKO SGN cell bodies showed increased membrane excitability [21], which at face value appear paradoxical since ABR PI amplitude is reduced and delayed (Figure 2). For 1-mo old SGNs, the most consistent findings were significant RMP depolarization in the DKO SGNs compare to WT neurons (apex; WT, -65 ± 3 mV, n = 31; DKO, -58 ± 2 mV, n = 27; *p* < 0.001: base; WT, -57 ± 2 mV, n = 25; DKO, -53 ± 4 mV, n = 31; *p* < 0.001), and reduced AP amplitude (apex; WT, 80.0 ± 2.7 mV, n = 31; DKO, 72.1 ± 4.8 mV, n = 27; *p* < 0.001: base; WT, 83.2 ± 3.4 mV, n = 25; DKO, 72.0 ± 5.1 mV, n = 25; *p* < 0.001; also see Figure 5). Although the DKO SGNs showed increased spike frequency compared to the WT, we observed an increased failure rate of evoked AP using suprathreshold current injection (Figure 4). Additionally, with constant subthreshold current injection, the extent of depolarization was more variable in the DKO than for WT neurons. As illustrated in Figure 4E–4H, the variance of membrane-potential jitters was greater for the DKO SGNs than age-matched WT neurons (Figure 4E, 4F). Increased membrane excitability and jitter in DKO SGNs were seen in 1, and 3-mo old mice. Surprisingly, the functional differences in response properties between the WT and DKO SGNs were not apparent in 4-5-mo old neurons (Figure 5). However, by 6 mos and older (9 mos), the response properties of basal SGNs from DKO compared to WT were in stark contrast to the increased membrane excitability shown in 1-3-mo old neurons (Figure 5). In older mice (> 6-mo-old), SGNs isolated from DKO mutant mice required increased current to evoke APs compared to age-matched WT SGNs (Figure 4I–4K). AP amplitudes, latency, and duration from both basal and apical neurons from older DKO mice were consistent with reduced membrane excitability; a startling transition from young (1-3-mo old) and robustly excitable electrophysiological phenotypes, in comparison with older SGNs (6-9 mo old) and relatively quiescent membrane activity (Figures 4, 5).

### The source of Na^+^ for K_Na_ channel activation

The Na^+^ source for activation of K_Na_ channels is expected to regulate the amplitude and kinetics of K_Na_ current profoundly. Since the major source of intracellular Na^+^ in SGNs is entry through voltage-gated Na^+^ current (I_Na_), we assessed the properties of I_Na_ in WT and DKO mice. In WT SGNs, under conditions where Ca^2+^, K^+^, and Cl^-^ currents were suppressed (see Methods), we observed ~-5-10 pA/pF (-7.1 ± 3.3 pA/pF, n = 34) holding current that was attenuated at potentials close to the reversal potential of Na^+^ (E_Na_). The holding current plummeted (-2.4 ± 1.6 pA/pF, n = 11; *p* = 0.001), using Na^+^ current recording bath solutions containing 1 μM TTX, suggesting the apparent persistent current was carried by Na^+^ channels, consistent with previous reports [18]. Another important observation was that in current-clamp mode, and using the regular bath and pipette solution, the RMP of SGNs shifted by ~5 ± 1 mV (n = 19) in the positive direction, upon application of 1 μM TTX (not shown).

**Figure 6:**
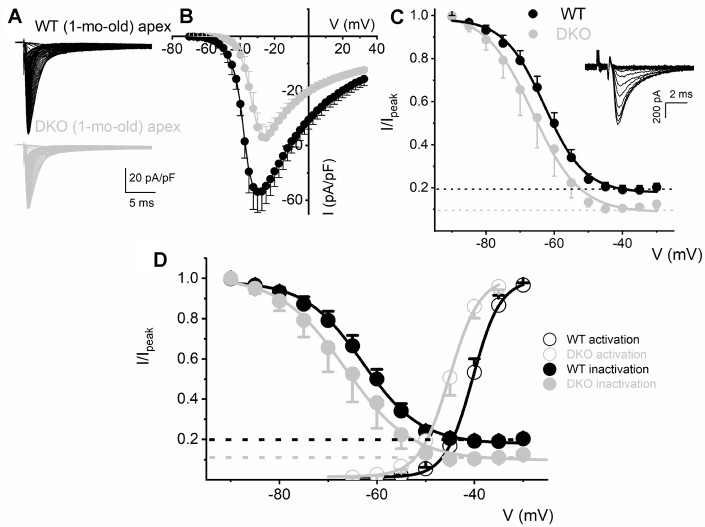
**Reduced Na^+^ current density and available current in K_Na_1 DKO SGNs.** (**A**) Whole-cell inward Na^+^ currents were elicited using depolarizing steps from -70 mV to 35 mV (ΔV = 2.5 mV). The currents were normalized to individual membrane capacitance (C_m_), were recorded from 1-mo-old mice using apical SGNs from WT (shown with black traces) and DKO (shown with grey traces). Data were generated from 19 SGNs from each experimental group. (**B**) We observed consistent differences in peak Na^+^ current (I)- voltage (V) relation among WT (●) apical and DKO (●) SGNs. (**C**) Voltage-dependent inactivation was tested at -10 mV, using pre-pulse voltages ranging from -90 mV to 60 mV (ΔV = 5 mV) (see representative traces, inset). The mean normalized peak recovered-current *versus* voltage relation for WT (black line and symbol) and DKO (grey line and symbol) SGN from apical third of the cochlea are plotted (n = 9). The half-inactivation potentials (V_1/2_) were measured by fitting plots to the Boltzmann function. The V_1/2_ for WT was -62.9 ± 2.5 mV and the slope factor *k*, was, 6.2 ± 0.7 mV (n = 11) and DKO was -66.3 ± 2.7 mV and the slope factor *k*, was 6.9 ± 0.3 mV (n = 13). (**D**, **E**) WT (**D**) and DKO (**E**) activation and inactivation curves to illustrate window current and sustained/persistent Na^+^ currents available for activation of K_Na_1 current. The activation curves for Na^+^ currents from WT and DKO were fitted with the Boltzmann function. The V_1/2_ for activation WT SGN was -40.4 ±3.6 mV, and the slope factor *k*, was 2.9 ± 0.5 mV (n = 9) and DKO was -45.1 ± 4.8 mV, and the slope factor *k*, was 3.08 ± 0.8 mV (n = 11).

**Figure 7:**
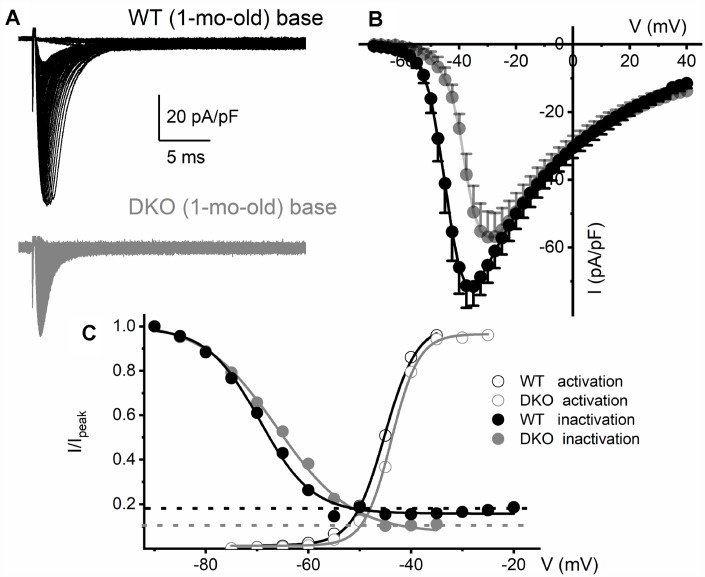
**Reduced Na^+^ current density and available current in K_Na_1 DKO SGNs.** (**A**) Whole-cell inward Na^+^ currents were elicited using depolarizing steps from -70 mV to 35 mV (ΔV = 2.5 mV). The currents normalized to individual membrane capacitance (C_m_), were recorded from 1-mo-old mice using basal SGNs from WT (shown with black traces) and DKO (shown with grey traces). Data were generated from 15 SGNs of each experimental group. (**B**) Consistent differences were noted in the peak Na^+^ current (I)- voltage (V) relation among WT (●) apical and DKO (●) SGNs. (**C**) In WT ~20% of the Na^+^ current is sustained, and in the DKO, ~10% persisted. Voltage-dependent activation and inactivation curves fitted with Boltzmann functions. Half-inactivation potentials (V_1/2_) were measured by fitting plots to them. The V_1/2_ of the steady-state inactivation for WT was -69.5 ± 3.1 mV and the slope factor *k*, was, 5.1 ± 0.9 mV (n = 9) and DKO was -66.3 ± 2.7 mV and the slope factor *k*, was 7.3 ± 0.7 mV (n = 11). The activation curves for Na^+^ currents from WT and DKO were fitted with the Boltzmann function. The V_1/2_ for activation WT SGN was -45.1 ± 2.8 mV, and the slope factor *k*, was 3.1 ± 0.8 mV (n = 9) and DKO was -43.8 ± 2.6 mV, and the slope factor *k*, was 2.7 ± 0.5 mV (n = 11).

To determine the biophysical underpinnings for the different evoked response properties of the WT and DKO SGNs, we measured whole-cell inward Na^+^ currents from WT and DKO SGNs from a holding potential of -90 mV, using depolarizing steps from -70 mV to 35 mV (ΔV = 2.5 mV). Inward Na^+^ currents, normalized to individual SGN membrane capacitance (C_m_), were recorded from apical and basal SGNs from 1-mo-old WT and DKO mice (Figures 6A, 6B; 7). We observed consistent differences in the peak current-density voltage relation (I-V) among WT apical (-57.2 ± 4.1 pA/pF) and basal (-71.2 ± 6.4 pA/pF), compared to DKO apical (-37.1 ± 3.7 pA/pF) and basal (-57.1 ± 3.9 pA/pF) SGNs, respectively. Voltage-dependent inactivation was tested at -10 mV, using pre-pulse voltages ranging from -90 mV to 60 mV (ΔV = 5 mV) (see representative traces, inset, Figure 6C). The normalized peak recovered-current *versus* pre-pulse voltage curves were generated from data collected in both 1-mo WT and DKO SGNs from apical and basal cochlear turns (Figures 6C, 7). Using 150-300-ms duration pre-pulses, ~20% of the WT and ~9% of the DKO Na^+^ currents appeared available for activation. The half-inactivation potentials (V_1/2_) and the slope factors for the inactivation curves were measured by fitting the plots to the Boltzmann function (summary data provided in legend, Figures 6 and 7). By superimposing the steady-state activation and inactivation curves of Na^+^ currents from WT and DKO SGNs, we estimated that ~20% of the WT current was available at the RMP. The sustained Na^+^ influx would activate the K_Na_ current compared to DKO current (~10%), which would be devoid of K_Na_ activation. The resultant activation of K_Na_ current in the WT is expected to shift the membrane voltage towards the reversal potential of K^+^ (E_K_ ~-80 mV). Indeed, the negative voltage shifts (5-8 mV) in the RMP are expected to increase the available evoked Na^+^ currents in both apical and basal SGNs, consistent with the data gleaned from TTX blockade of the Na^+^ current. These results are in keeping with the significant increase in AP amplitude and rapid upstroke (dV/dt) of WT neurons (Figure 4A). In contrast, in DKO SGNs, depolarized membrane voltage coupled with reduced availability of Na^+^ current promotes membrane jitters and AP failure, despite the closeness of the RMP of DKO SGNs to AP threshold.

### Intracellular Ca^2+^ handling is altered in SGN from DKO mice

**Figure 8:**
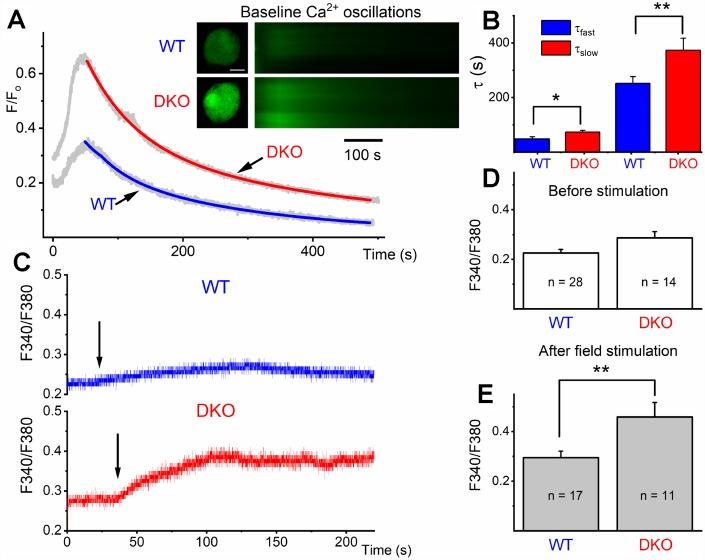
**Ca^2+^ transients in 1-mo old SGNs from WT and DKO mice.** (**A**) Representative examples of line-scan images (inset) captured from unstimulated but spontaneous Ca^2+^ oscillations showing Ca^2+^ transients recorded from WT (upper panel, in black) and DKO (lower panel, in gray) SGNs. Sample records show SGN loaded with Fluo 4. (**B**) Summary data for the fast and slow time constant (τ_1_ and τ_2_) of the Ca^2+^ transient decay at baseline using two exponential functions. The τ_1_ (ms) for WT = 48.4 ± 7.9; DKO = 73.2 ± 6.3; n = 19 (*p* = 0.019): and τ_2_ (ms) for WT = 251.7 ± 24.5; DKO = 373.3 ± 44.2; n = 17 (*p* = 0.022). (**C**) Sample records show a region of interest (ROI) of ratiometric Fura 2 assessment of field potential (10V/4Hz) depolarization of WT and DKO SGN (arrows show the time of stimulation). Recovery after field stimulation had ~500-1000s time course (not shown). (**D**, **E**) Summary data for the amplitude of the total Ca^2+^ at baseline and after field stimulation in WT and DKO SGNs (1-mo old apical neurons). The F_340/380_ for WT before field stimulation = 0.23 ± 0.01 (n = 28); DKO = 0.29 ± 0.03; n = 14 (*p* = 0.029): and after field stimulation WT = 0.29 ± 0.03 (n = 17); DKO = 0.46 ± 0.06; n = 11 (*p* = 0.007). Significant differences between genotypes were determined using unpaired two-tailed t-test.

The transition from depolarized to hyperpolarized membrane potential from 1 and 3 mo to 6 and 9 mo old DKO SGNs suggested potentially relevant intervening compensatory changes. To begin to address the cellular mechanisms, we tested whether the initial enhanced membrane depolarization in the DKO SGNs may promote increased Ca^2+^ influx compared to age-matched WT neurons. We monitored intracellular Ca^2+^ concentration in 1-mo old WT and DKO SGNs using the Ca^2+^ indicator Fluo-4, and ratiometric Ca^2+^ (Fura-2) imaging. We measured field-potential evoked Ca^2+^ transients in SGNs loaded with fluo-4. The Ca^2+^ transients were captured using confocal line scan imaging (Figure 8A). The decay phase of the Ca^2+^ transients was fitted using two exponential functions (Figure 8B), showing a significant prolongation in DKO neurons. Ca^2+^ transient amplitude was also significantly increased in DKO SGNs.

Increased Ca^2+^ levels could affect overall Ca^2+^ homeostasis. To substantiate these findings, we used ratiometric Ca^2+^ dye, Fura-2, for semi-quantitative assessment. SGNs cell bodies loaded with Fura-2 AM were imaged. The cell bodies of 1-mo old WT and DKO SGNs had a relative baseline Ca^2+^ concentrations that were significantly different (F340/F380; WT = 0.226 ± 0.03 (n = 28), DKO = 0.286 ± 0.025 (n = 14; *p* < 0.05: Figure 8C, 8D). Field stimulation of Fura-2 AM loaded SGNs isolated from DKO showed ~2-fold Ca^2+^ rise (Figure 8C, 8E). The slow delay in recovery after baseline rise in Ca^2+^ and increased amplitude of Ca^2+^ transient is likely to alter Ca^2+^ handling in DKO SGNs compared to WT neurons, resulting in a long-term rise in Ca^2+^ concentrations. These intrinsic alterations in Ca^2+^ handling are expected to have short- and long-term effects on synaptic and neuronal functions.

### Delayed synaptopathy and normal SGN myelination in DKO mice

**Figure 9:**
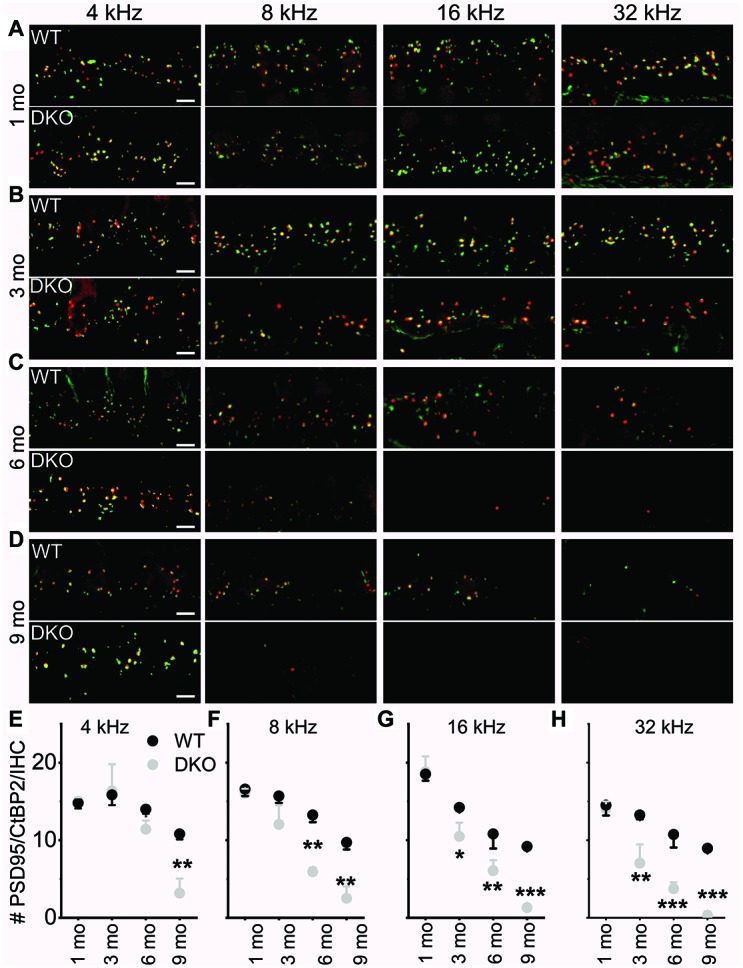
**Presynaptic and postsynaptic marker counts are normal in K_Na_1 DKO in 1-3-, but not 6-9-mo old mice.** Synapses between the spiral ganglion neurons (SGNs) and inner hair cells (IHCs) were quantified at four tonotopic locations (4, 8, 16, and 32 kHz) in the organ of Corti isolated from 1-9-mo old WT and DKO mice. (**A**–**D**) There were no obvious differences between WT and DKO mice in the organization of afferent synapses, identified as paired CtBP2 (red) and PSD95-(green) immunopuncta. Images presented as Z-projections were made using stacks of confocal micrographs from the 4, 8, 16, and 32 kHz region as indicated. (**E**–**H**) Quantification of the average number of synapses per IHC showed no statistically significant differences between WT (black) and DKO (grey) animals at 1 month of age. However, with increasing age from 3- to 9-mo, statistical differences among the synapse counts appeared and gradually encompassed more tonotopic regions. Statistical differences were observed first at the high-frequency segment of the cochlea. Values (mean ± SEM) are illustrated (*p* < 0.05, 0.01, 0.001 = *, **, ***). Scale bar = 3 μm.

**Figure 10:**
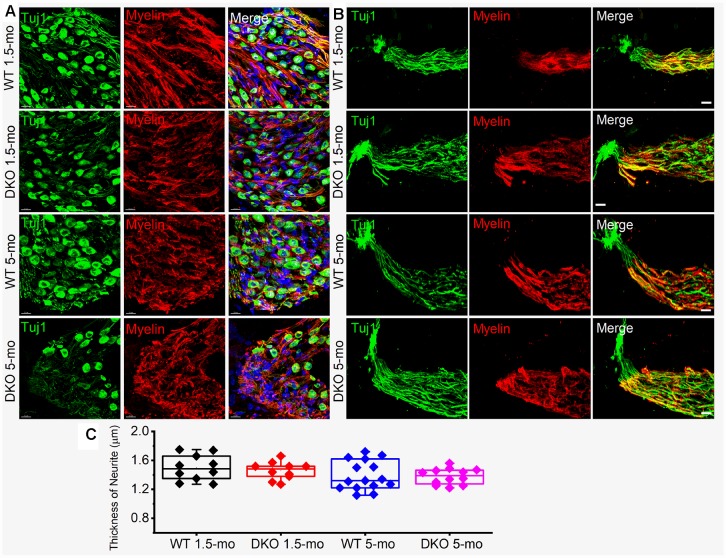
**Myelin density and neurite thickness are unaltered in 1.5-5-old WT and DKO mice.** (**A**) Sections of 1.5- and 5-mo-old WT and DKO spiral ganglion (SG) labeled with Tuj1 (green), and myelin (red) antibodies. Sections were obtained from the basal region of the cochlea. (**B**) Sections of 1.5 and 5-mo old WT and DKO peripheral neurites of SGN labeled as in (**A**). (**C**) Box plot of neurite thickness (diameter) at different age groups. Scale bar = 10 μm.

The existing correlation in HHL is a loss of afferent synapses between IHC and SGNs and demyelination of SGN dendritic projections [3, 8], while overt hearing loss is ascribed to HC or SGN loss [49–51]. Using N-terminal antibodies to CtBP2 and PSD95, we performed immunostaining for presynaptic ribbons and postsynaptic receptors in WT and DKO mice. Although HHL was observed in 1 and 3 mo old DKO mice, IHC synaptic density was similar between WT and DKO cochleae in all frequency regions examined (Figure 9A, 9B, 9E–9H). By contrast, DKO mice older than 6-mos old showed a robust reduction in IHC synaptic density compared to their age-matched WT controls (Figure 9C, 9D, 9E–9H). Next, we used anti-myelin immunostaining to assess potential changes in SGN myelination. Semi-quantitative analyses of myelin and axonal thickness along the peripheral axons did not show statistical differences between WT and DKO mice before the onset of increased ABR thresholds (Figure 10; 1.5 mos (thickness in μm), WT = 1.5 ± 0.18 (n = 10), DKO = 1.5 ± 0.12 (n = 10); *p* = 0.56; 5 mos, WT= 1.4 ± 0.20 (n = 15), DKO = 1.4 ± 0.11 (n = 12); *p* = 0.89).

**Figure 11:**
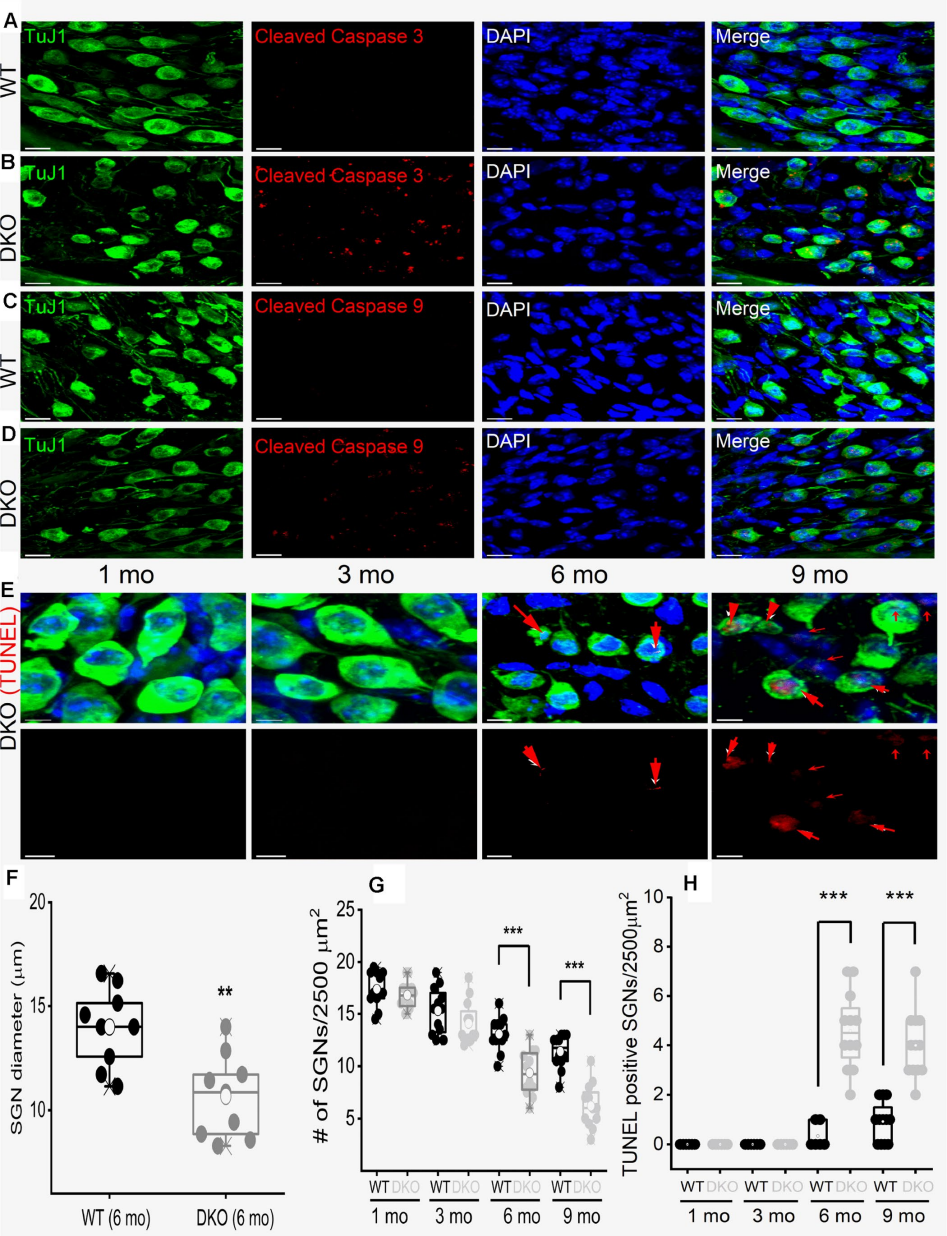
**Age-related degeneration of SGN through apoptosis signal.** (**A**–**D**) Immunofluorescence detection of cleaved caspase 3 (red; **A**–**B**) and caspase 9 (red; **C**, **D**) in WT and DKO 5-mo old cochlear sections. SGNs were labeled with neuronal marker TuJ1 (green). The nuclei were stained with DAPI (blue). Scale bar: 20 μm. Increased active caspase 3/9 was seen in DKO cochlear sections. Scale bar = 10 μm. (**E**) Shown are cochlear sections of in DKO cochleae at difference ages (1-9 mos) assessed for with TUNEL assay (TUNEL-positive in red). Scale bar: 5 μm. (**F**) Summary histogram showing a significant reduction in SGN size (diameter) between WT and DKO mice at 6-mo old (data from 9 mice each). (**G**) Age-dependent (1-9 mo) reduction in SGN densities (data from 8-9 mice each). Comparison between WT and DKO mice. (**H**) Summary data from WT and DKO cochleae at ages indicated, showing increased TUNEL-positive SGNs in 6-9–mo old (data from 8-9 mice each). Significant differences between genotypes were determined using unpaired two-tailed t-test (* *p* < 0.05, ** < 0.01, *** < 0.001).

To account for SGN loss, we stained neurons with TUJ1 antibodies and sampled neuronal numbers from 1- to 9-mo-old mice. Synaptic and SGN loss was seen in 6-mo and older mice (Figure 11). SGN degeneration was preceded by a significant reduction in the soma size, as illustrated by measuring neuronal diameters (Figure 11). The demonstrable rise in Ca^2+^ can mediate SGN degeneration through multiple cell-death mechanisms [75, 39, 42]. To determine whether SGN loss found in the DKO mice resulted from apoptotic cell death, we used the TUNEL assay to determine neuronal survival (Figure 11). Using an antibody that specifically recognizes the cleaved form of caspase-3 (17 kDa), we analyzed sections of the SG of WT and DKO cochleae and detected caspase-3 activation by 5-mo of age (Figure 11A, 11B). There was no detectable cleaved caspase-3 in the SGN from 9-mo-old WT mice. Using an antibody that recognizes explicitly cleaved caspase-9, the activated 17-kDa fragment was detected in 5-mo old DKO mutant compared to WT SGNs (Figure 11C, 11D). Downstream caspases can be activated in the presence and absence of caspase-9 cleavage [43]. Increased TUNEL-positive SGN numbers were observed in 6-9 mo old DKO mice, as documented in the summary histograms (Figure 11E, 11H). For clarity in visualizing TUNEL-positive SGNs, we show photomicrographs without neuronal markers below (Figure 11E). Activation of caspases is transient and precedes apoptotic cell death. Induction of cleaved and activated caspases in the WT and DKO mutant SGNs were studied at survival times that preceded the robust loss of neurons (5 mo). The results raised the possibility that there might be stepwise transitions that culminate in morphological changes and neuronal loss in the DKO mice.

### SGN degeneration may be correlated with altered Ca^2+^ and inositol 1,4,5 trisphosphate receptor type 1 (IP_3_R1) signaling

**Figure 12:**
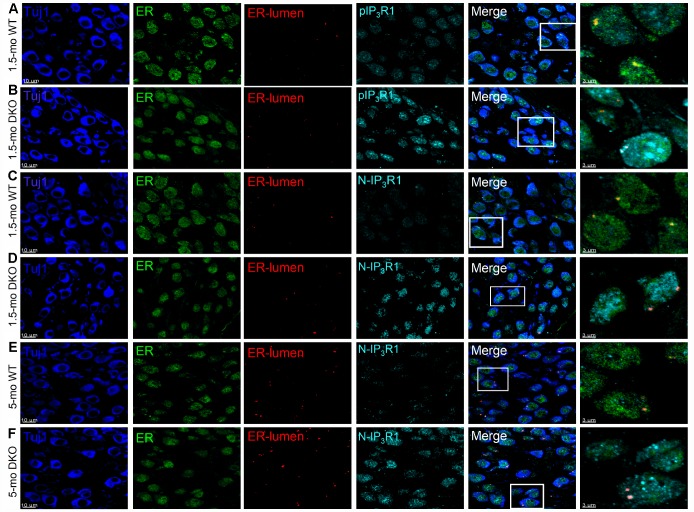
**Detection of IP3R1, pIP3R1, and** its **proteolytic fragments in SGNs from WT and DKO cochlea.** (**A**–**F**) Immunofluorescence detection of the endoplasmic reticulum (ER)-lumen domain of IP3R1(red), phosphorylation site (pIP3R1) (cyan; **A**–**C**), N-terminal domain (N-IP3R1) (cyan; **C**–**F**). We examined basal cochlear sections at 1.5-mo and 5-mo WT and DKO mice. SGNs were labeled with neuronal marker TuJ1 (blue). The ER was stained with ER-marker (blue). Merged images are shown together with digitally magnified (~3X) images, shown in the last panel. Scale bar 3 μm. For pIP3R1 at 1.5 mos, the percent of SGNs with positive reactivity for WT was 12 **±** 3, and DKO was 54 **±** 9; (p < 0.0001, data from 7 mice obtained from 25 section/mice). N-IP3R1 at 1.5- and 5-mos, percent cell reactivity for WT = 6 **±** 3; DKO = 37 **±** 12, and WT = 9 **±** 4; DKO = 61 **±** 12, respectively (p < 0.0001, data from 6 mice obtained from 25 section/mice). Scale bar = 10 μm.

Increased Ca^2+^ transients, as well as a delay in the bi-exponential kinetics of Ca^2+^ dynamics as observed in DKO SGNs (Figure 8), may be associated with overall alterations of Ca^2+^ homeostasis, including Ca^2+^ release from intracellular sources. Therefore, we determined whether altered IP_3_R1 regulatory mechanisms were involved in DKO SGN Ca^2+^ elevation compared to age-matched WT neurons, using IP_3_R1-specific antibodies to detect specific fragments of the protein (Figure 12), including 1) endoplasmic reticulum (ER) lumen region, 2) phosphorylation-site (pIPR1), 3) N-terminal binding (NIP_3_R1), and 4) cytoplasmic epitope (cleavage site overlap, IP_3_R1). Compared to WT SGNs, the levels of phosphorylated IP_3_R1 (pIP_3_R1) and N terminal fragment of IP_3_R1 (NIP_3_R1) were higher in the DKO at 1.5-mo and 5-mo old (Figure 12). Of note, by 5-mo, the levels of pIPR1 in WT and DKO SGNs were similar (not shown). However, qualitative assessment of antibody reactivity to the cytoplasmic epitope (1883 ~ 1902) that should react with the total IP_3_R1 suggested that the overall IP_3_R1 levels remain relatively unchanged (not shown). The results raise the possibility that DKO SGNs have increased N-terminal cytoplasmic fragments. Cleaved-IP_3_R1 (“leaky receptor”) is subjected to unregulated Ca^2+^ release that likely increases Ca^2+^ levels in DKO SGNs. However, further studies are required to demonstrate direct association between the rise in intracellular Ca^2+^ and IP_3_R1 signaling.

## DISCUSSION

Peripheral axons/dendrites have segmental specializations, such as heminodes and node of Ranvier, interspersed with myelinated internodes for the initiation and saltatory conduction of APs. These vital roles are mediated by clustered subcellular expression of voltage-dependent Na^+^ and K^+^ channels at these privileged excitable domains [44, 45]. The importance of these ion channels and their site-specific location, as well as myelin in nerve conduction, is underpinned by neurological diseases, such as epilepsy [46] and multiple sclerosis [47], which are aligned with their mutations and demyelination. As it turns out, several of these diseases share HL as one of their symptoms. In the present study, we have discovered the axonal localization of K_Na_ channel transcripts and identified a new role of K_Na_1 current, namely that this current is partially activated at rest (~20%) and is sustained at the RMP.

In contrast to the expected attenuation of a K^+^ current in membrane excitability, the resting activity of K_Na_1 in SGN enhances the availability of the transient Na^+^ current, increasing the upstroke phase of APs. Additionally, the interplay between the Na^+^ and K_Na_1 currents reduces membrane jitters in AP fidelity and delays, ensuring invariant timing of APs. The resulting altered AP phenotypes are expected to improve conduction speed and spike reliability [19], a requisite for normal auditory processing [35]. We demonstrated that the genetic ablation of *Kcnt1/2* resulted in HHL that transitions gradually to overt HL, reminiscent of human HL [48]. In this model of *Kcnt1/2* deletion, increased membrane excitability culminates with a gradual rise in intracellular Ca^2+^ and disruption in cytoplasmic Ca^2+^ handling. We propose that these changes may precipitate activation of caspases 3 and 9, leading to proteolytic fragmentation of IP_3_R1 and further activation of caspases to mediate apoptosis and resulting in SGN synaptic and somatic degeneration.

### Robust expression of *Kcnt1/2* transcripts and proteins in the axons and cell bodies of SGNs but not in hair cells

The expression of K_Na_1 is not restricted to SGNs and has been identified in the central AN, such as the MNTB, where K_Na_ currents regulate spike timing [24, 25]. It is highly unlikely that the central role of the K_Na_ current is responsible for the observed ABR peak I amplitude reduction and delayed latency, and the HHL, ensuing from the null deletion of *Kcnt1* and *Kcnt2*. First, changes in ABR peak I latencies are impervious to altered central AN activity [49]. Second, alterations in the activities of neurons in the CN at the level of the MNTB are likely to affect peak III amplitudes and latencies. Third, both experimental and computational analyses have shown that ABR peak I originate exclusively from hair cells and SGN [38, 50]. Last, mature hair cells do not express functional Na^+^ currents, and in cases where the current has been identified, the voltage-dependent activation is extremely negative and falls outside the voltage ranges of the RMP of hair cells [51]. Therefore, K_Na_ currents, if present in hair cells, are unlikely to have a functional impact. Furthermore, we did not find *Kcnt1/2* transcripts and proteins in hair cells, ruling out the possibility that the observed HHL originated from hair cell dysfunction.

In contrast, SGNs show robust expression of *Kcnt1/2* transcripts and channel proteins in the axons and cell bodies. Moreover, recent studies [21] and the present findings strongly suggest that the activity of K_Na_1/2 channels is essential for primary auditory functions. Thus, the most plausible explanation for the observed results is altered SGN functions, including synaptic, axonal conduction, and somatic dysfunction and degeneration.

### RNA composition of subcellular compartments

Subcellular domain-specific RNA expression suggests enduring cellular processes occurring within that compartment. The use of RNA deep sequencing and high-resolution hybridization analyses have identified a plethora of mRNA species in neuronal axons and dendrites, including hippocampal CA1 neuropil, changing the prevailing notion of the capacity for mRNA localization and local protein translation [52–55]. Found predominantly as a developmental occurrence in the dynamic process at growth cones, β-actin and other structural protein mRNA were localized and translated in response to netrin I gradient in growth cones [56, 57]. Axoplasm extracts from giant axons have also been found to contain ribosomal RNA and transcripts that encode for protein-synthesis machinery proteins, indicating that local protein synthesis is a basic property of axons [33, 58, 59]. Indeed, axonal transcriptome from motoneurons and sensory neurons all contain membrane protein mRNA, including K^+^ channels [1, 60]. To our knowledge, this is the first report to demonstrate the expression of membrane protein transcripts encoding *Kcnt1* and *Kcnt2* in primary ANs. Consistent with the present report, immunostaining studies substantiated the expression of the channel proteins in the axolemma; although at present, we have no evidence to preclude that membrane protein, which is translated at the cell bodies, are shuttled to the axons. Nonetheless, our data suggest that SGNs may use structural and local domain-specific protein translation to confer multiple functions as described in central and peripheral neurons [1].

### Multifaceted mechanisms of HHL

Hair cell-SGN afferent synaptic defects are one of the hallmarks of HHL. They are represented as degraded AN amplitude and latency responses and marked by pre- and post-synaptic marker mismatch and reduced synaptic densities [8, 12]. Disruption of myelination of peripheral axons of SGNs can also lead to HHL [3]. The present study suggests that there are no measurable changes in synaptic densities from the inception and the duration of the HHL phenotype in 1 and 3 months of age.

Additionally, using semi-qualitative and structural assessment of myelination between the WT and DKO mice SGNs, we did not identify any altered axonal myelination. The results argue against the contribution of structural defects in the underlying mechanisms of HHL in the mouse model. Instead, increased membrane jitters, reduced availability of Na^+^ currents and the resulting decline in the dV/dt of AP upstroke, and increased variability in spike timing introduced by ablation of K_Na_ currents, are the likely functional aberrations, leading to the HHL phenotype. We interpret single AN electrophysiologic changes with great caution. Extending single-neuron properties to explain the ABR phenotype may be an oversimplification. However, it is reasonable to expect that the ensemble average of single neuronal activity represents the overall AN response property.

The role of K_Na_ current in reducing membrane noise and the increased membrane jitters in the DKO SGNs can result in AP conduction failure and delays [19]. These are consistent with changes in peak I latencies between the WT and DKO ABR. Outward current mediated by K_Na_ channels contributes to the setting of the RMP of SGNs. Activation of K_Na_ current mediates membrane hyperpolarization that indirectly influences the steady-state available Na_v_ channels. Increasing AP peak amplitude from membrane hyperpolarization is also expected to drive Ca^2+^ influx, regeneratively increasing the AP overshoot. Thus, a reduction in AP amplitude after the deletion of K_Na_ channels dovetail well with the present report. Peak I amplitude of the ABR waveform originates from multiple sites of type I SGN afferents, including the unmyelinated post-synaptic terminal, the spike generator heminode [61], and saltatory conduction nodes of Ranvier. We propose that the resulting AP dyssynchronization and amplitude reduction, upon deletion of K_Na_ current, are the underlying mechanisms for the loss of both amplitude and temporal resolution of the peak I ABR waveform. Since the peak I of the ABR waveform represents a gross afferent SGN discharge that originates from multiple sources and HHL is defined by an altered peak I profile, it is conceivable that there is a multifaceted etiology.

### The transition from HHL to overt HL

Ca^2+^ has at least two competing effects on SGN functions. Moderate rises in Ca^2+^ promote SGN survival *in vitro* [62], whereas a sustained increase in Ca^2+^ mediates degenerative processes, including apoptotic cell death [75]*.* The findings suggest that K_Na_ current, through sustained Na^+^ influx, draws the RMP towards hyperpolarized potentials, E_K_, which in turn increases available Na^+^ current for activation. K_Na_ current attenuation likely mediates sufficient membrane depolarization of the RMP, reducing available Na^+^ current, which reduces not only the AP amplitude but also the rate of change of membrane voltage. We propose that sustained membrane depolarization in DKO SGNs may produce increased Ca^2+^ influx, triggering long-term aberrant Ca^2+^ handling, activation of caspase 3/9, and IP_3_R1 dysfunction and apoptotic neuronal degeneration. Indeed caspase 3 activation-mediated SGN degeneration has been demonstrated, and inhibition of caspase activity suffices to attenuate HL in mouse models [63]. Conformational changes ensuing from IP_3_ binding to IP_3_R1 mediate channel gating to release Ca^2+^ from the ER [64]. Proteolytic fragmentation of segments of the N-terminal cytoplasmic domain may favor prolonged open state, resulting in ER Ca^2+^ leakage that reinforces a rise in cytosolic Ca^2+^ [65].

Ion channel activity and changes in ion concentration are inexplicably linked to cellular viability and apoptotic cell death. Increased intracellular Ca^2+^ induces mitochondrial-dependent apoptosis [66, 67]. We show that altered Ca^2+^ level induced activation of caspase 3/9. Ca^2+^ and caspase-mediated cleavage of phosphorylated IP_3_R1 and truncated IP_3_R1 result in Ca^2+^ leakage to the cytoplasm from the ER. Increased cytosolic Ca^2+^ exerts positive feedback, stimulating mitochondria-mediated increase cleaved caspases [65, 68]. The findings provide definitive evidence to implicate the underlying Ca^2+^ dysfunctional cascade as a potential platform for SGNs degeneration. However, what remains unsolved is the complex interaction between Ca^2+^-dependent caspase activation and the initiation of the SGN degenerative process. Additionally, because Ca^2+^ signaling is at a bifurcation point between plasticity and cell death, the challenge is to determine the levels of Ca^2+^ and conditions that favor one of these major crossroads of neuronal activity for therapeutic intervention.

It is conceivable, albeit unlikely that potential compensatory changes ensuing from the null deletion of *Kcnt1* and *Kcnt2* may mask generalization of the findings on this report. The C57 background strain used as WT control belongs to a group of mouse strains that exhibit inherent ARHL [69]. Although the underlying mechanisms for the ARHL in mice have been assigned to the *ahl1* allele, which represents cadherin 23 (hair bundle protein) mutation [70], using a WT control without this caveat (e.g., CBA/CaJ mouse model) would have been preferred. Also, direct synaptic recordings to determine K_Na_ current functions in SGNs would substantiate conclusions gleaned from the present study. Finally, the interplay between Na^+^ and Ca^2+^-dependent regulations in ANs inferred from the present findings requires further examination.

## MATERIALS AND METHODS

The Animal Care and Ethical Committee of the University of Nevada Reno approved the use of animals in this study. All mice were bred in-house under a 12:12 h light-dark cycle. Wildtype (WT) and *Kcnt1^-/-^/Kcnt2^-/-^* (double knock out mutant (DKO mutant)) mice ages (1 to 9-months old) were used. Breeding pairs of the DKO mutants and the WT control mice were obtained from the laboratory of Dr. S. Pyott (University of Groningen). However, the mice originated from the laboratory of Dr. C. Lingle (Washington University, St. Louis) [23]. K_Na_1 double knockout (DKO) mice were bred onto a C57BL/6 background for 12 generations [23]. The utilization of wildtype (WT) littermates was not feasible. Thus, age and gender-matched C57BL/6 mice were obtained from the Jackson Laboratory and used as WT controls. The whole-genome scanning of the C57BL/6 (C57) and the DKO sub-strains was performed as described previously [21].

### Auditory brainstem responses (ABR)

At least twelve WT, and ten DKO mutants, mice (grouped into age ranges; 1-, 3-, 6- and 9-month old) were anesthetized with ketamine (100 mg/kg) and xylazine (20 mg/kg) prior to recordings, and ABR measurements were recorded as described previously [71, 72]. Briefly, a ground needle electrode and recording needle were placed subcutaneously in the scalp, and a calibrated electrostatic speaker coupled to a hollow ear bar situated inside the external ear. Broadband clicks and pure tones (4, 8, 16, and 32 kHz) were presented in the animal’s ear in 10 dB increments, starting from 0 dB SPL and ending at 100 dB SPL. The ABR sweeps were computer-averaged (time-locked with the onset of 512 stimuli, at 20/s) out of the continuous electroencephalographic activity. The threshold of the hearing was determined as the lowest intensity of sound required to elicit a characteristic waveform. Peak (P)I and PII were detected manually in a blind faction to genotype and clicked sound and tone pips, to calculate wave I and II amplitudes and latencies. Input-output (I/O) function slopes of the amplitude and latency growth function curves were calculated as described previously [73] over stimulus intensities ranging from 10-100 dB SPL. I/O function slopes were determined when distinct positive and negative peaks could be identified unmistakably.

### Single-molecule fluorescence in situ hybridization (smFISH) with RNAscope

Mice were anesthetized with an intraperitoneal injection of ketamine (100 mg/kg) and xylazine (20 mg/kg) and were transcardially perfused with diethyl pyrocarbonate (DEPC)-treated phosphate buffer saline (PBS) and 4% paraformaldehyde (PFA) in DEPC-treated-PBS to preserve RNA. Tissue samples from cochleae were harvested and kept in a 4% PFA in DEPC-treated solution, overnight on a shaker at 4°C. Samples were washed with DEPC-treated-PBS, and cochleae were decalcified in 10% ethylenediaminetetraacetic acid (EDTA) in DEPC-treated-PBS for 1-4 days, depending on the age of mice, on a shaker at 4°C and washed with DEPC-treated-PBS. Samples were sequentially dehydrated in 10%, 20%, and 30% sucrose solution at 4°C for 1 hr, 2 hrs, and overnight, respectively. Samples were transferred into optimal cutting temperature (OCT) compound for a minimum of 1 hr at 4°C and then snap frozen, using a dry ice-ethanol mixture. Samples were cryo-sectioned to a thickness of 14 μm, placed onto superfrost plus microscope slides, and stored at -80 °C until processed.

Probe hybridization was performed according to the manufacturer’s instructions (Advanced Cell Diagnostics, Newark, CA). Sections were immersed in pre-chilled 4% PFA for 15 min at 4°C. Sections were then dehydrated at room temperature (RT) in 50%, 70%, and twice in 100% ethanol for 5 min each and allowed to dry for 1-2 min. Fixation and dehydration were followed by hydrogen peroxide reaction for 10 min at room temperature (RT) then, protease digestion, using protease 4 for 30 min at RT. Sections were then incubated at 40°C with the following solutions: 1) target probe in probe diluent buffer for 2 hrs; 2) preamplifier in AMP 1 for 30 min; 3) amplifier in AMP 2 for 30 min; and 4) conjugation with dye-labeled probe in AMP 3 for 15 min; 5) the appropriate HRP-channel for 15 min; 6) Opal dye for fluorescent detection for 30 min; and 7) HRP blocker for 15 mins. After each step, slides were washed with buffer two times for 2 min at RT. Probes for K^+^ channels and glyceraldehyde 3-phosphate dehydrogenase (*Gapdh)* positive and blank negative controls were obtained from ACD. Sequences of the target probe, preamplifier, amplifier, and label probe are proprietary. Detailed information about the probe sequences can be obtained by signing a nondisclosure agreement provided by the manufacturer.

For subsequent immunofluorescent staining, slides were treated with 10% blocking solution for 30 min at RT, incubated with primary antibody (anti-β-III tubulin, TUJ1, Bio Legend, 1:500 dilution), overnight at 4°C, washed with TBS-0.005%Tween20 three times for 5 min each, incubated with secondary antibody (Alexa 594 and Alexa 647, Life Technologies, 1:500) for 2 hr at RT, and again washed with TBS-0.005%Tween20 three times for 5 min each. Incubation in DAPI solution for 30 s at RT was performed to label cell nuclei. Slides were then mounted in Fluoromount-G and sealed under a coverslip.

### Immunofluorescence

Cochleae were dissected from the temporal bone and fixed in 4% PFA in PBS for 2 hr and processed sequentially with 10% EDTA for decalcification. Cochleae samples were sequentially dehydrated in 10%, 20%, and 30% sucrose solution at 4°C for 1 hr, 2 hr, and overnight, respectively, then embedded in OCT for cryosectioning in the modiolar plane. Sections of 10 μm were washed in PBS, permeabilized in PBST (0.1% Triton X-100 in PBS) for 25 min, and then incubated for 60 min in a blocking solution containing 1% bovine serum albumin (BSA) and 10% goat serum in PBST. The sections were incubated with each primary antibody in an antibody solution containing 3% goat serum in PBS overnight at 4°C. The rinsed sections were then incubated for 2 hr at RT in a fluorescent dye-conjugated secondary antibody. The following primary antibodies were used: rabbit anti-KCNT1, KCNT2 (Alomone, Jerusalem, Israel), rabbit anti-cleaved caspase-3 and -9 (Cell Signaling Technology, Danvers, MA), rabbit and mouse anti-myosin 7a (Proteus Biosciences, Novus Biological and Developmental Studies Hybridoma Bank (DSHB)) mouse anti-PSD95 (Millipore), mouse anti-CtBP2 (BD Biosciences, San Jose, CA), rabbit anti-active-caspase-9 and rabbit anti-active-caspase-3 (Abcam, Cambridge, MA), rabbit anti-N-IP_3_R1 (Biorbyt, San Francisco, CA) rabbit anti-pIP_3_R1 (Invitrogen, Carlsbad, CA), rabbit anti-IP_3_R1 (cleavage site, Novus Biologicals, Centennial, CO), rabbit anti-ER lumen IP_3_R1 (Sigma), rabbit anti-calnexin (ER marker, Abcam) and mouse anti-neuronal class III ß-Tubulin (TUJ1) (Covance, Princeton, NJ). Secondary antibodies were Alexa Fluor 488 goat anti-mouse IgG2a, Alexa Fluor 568 goat anti-mouse IgG1 (Life Technologies, Carlsbad, CA), fluorescein (FITC)-conjugated affinity-purified goat anti-rabbit IgG, Alexa Fluor 647 affinity-purified goat anti-mouse IgG, and Cy3-conjugated affinity-purified goat anti-mouse IgG (Jackson ImmunoResearch Laboratories, West Grove, PA). Images were captured with a Nikon A1 and Olympus FV1000 confocal microscope.

### Isolation of SGNs and electrophysiological recordings

SGNs were isolated from male and female WT and DKO mice, as described in detail previously [74–77]. Mice were euthanized in a CO_2_ chamber, and the temporal bones were removed in a solution containing Minimum Essential Medium with Hank’s salt (Invitrogen), 0.2 g/L kynurenic acid, 10 mM MgCl_2_, 2% fetal bovine serum (FBS; v/v), and 6 g/L glucose. The SGN tissue was dissected and split into three equal segments: apical, middle, and basal turns across the modiolar axis. Apical and basal tissues were used to obtain viable neurons for patch-clamp experiments. Tissues were pooled from three mice into each SGN culture. Apical and basal tissues were digested separately in an enzyme mixture containing collagenase type I (1 mg/mL) and DNase (1 mg/mL) at 37 °C for 20 min. After a series of gentle trituration and centrifugation in 0.45 M sucrose, the cell pellets were reconstituted in 900 μl culture media (Neurobasal-A, supplemented with 2% B27 (v/v), 0.5 mM L-glutamine, 100 U/mL penicillin; Invitrogen) and filtered through a 20- μm cell strainer for cell culture. SGNs were cultured for 24 to 48 hrs to allow detachment of Schwann cells from neuronal membrane surfaces. All electrophysiological experiments were performed at RT (21-22°C). Tetrodotoxin (TTX) was applied at a perfusion rate of 1 ml/min for 5 min at a concentration of 10-1000 nM to block Na^+^ currents. Chemical reagents were obtained from Sigma-Aldrich (St. Louis, MO) unless otherwise noted**.** Other ionic currents such as K^+^ and Cl^-^ currents were suppressed using K^+^ channel blockers and ion substitution.

### Current and voltage-clamp experiments

Whole-cell membrane potential recordings were performed using an Axopatch 200B amplifier (Molecular Devices, Sunnyvale, CA). Membrane potentials were amplified, bandpass filtered (2-10 kHz), and digitized at 5-50 kHz using an analog-to-digital converter (Digidata 1200, Molecular Devices) as described earlier [78, 79]. Electrodes (2-3 MΩ) were pulled from borosilicate glass pipettes, and the tips were fire-polished. The normal extracellular/bath solution consisted of (in mM) 130 NaCl, 5 KCl, 1 MgCl_2_, 2 CaCl_2_, 10 D-glucose, and 10 4-(2-hydroxyethyl)-1-piperazineethanesulfonic acid (HEPES), pH 7.3. The normal internal/pipette/ solution contained (in mM) 132 KCl, 1 MgCl_2_, 0.01 CaCl_2_, 2 ethylene glycol-bis(β-aminoethyl ether)-N,N,N′,N′-tetraacetic acid (EGTA) 5 ATP-K_2_, and 10 HEPES, pH 7.3. The seal resistance was typically 5-10 GΩ. Capacitance and series resistance compensation (>90%) were made, and traces were filtered at 2 kHz using an 8-pole Bessel filter and sampled at 5 kHz. The liquid junction potentials (LJP) were measured (2.1 + 1.2 mV; n = 87) and corrected as described previously [80]. Data analyses were performed using the pClamp and Origin software (MicroCal Inc., Northampton, MA). Where appropriate, pooled data are presented as means + S.D.

Whole-cell voltage-clamp recordings were conducted at RT, using an Axopatch 200B amplifier and filtered at 2 kHz through a low-pass Bessel filter. Data were digitized at 0.5-1.0 kHz using a Digi-data analog-to-digital converter. SGNs, after 1-2 days in culture, was held in a bath solution (in mM; 4 KCl, 2 MgCl_2_, 0.1 CaCl_2_, 10 HEPES, 10 D-glucose, 75 NaCl, 50 N-methyl glucamine, 20 tetraethylammonium chloride (TEACl), and 5 4-Aminopyridine (4-AP)). The bath solution composition curbed K^+^ currents. We suppressed Ca^2+^ and Cl^-^ currents by using 0.05 mM bath Ca^2+^ and replacing 90 mM bath Cl^-^ and pipette solution with aspartate. Replacing Cl^-^ with aspartate increased the LJP by 9.5 + 1.8 mV (n = 36), which was corrected. Borosilicate glass pipettes were pulled using a Sutter P-97 Flaming-Brown micropipette puller (Sutter Instruments) and fire-polished for an optimal pipette resistance (2-3 MΩ). Pipettes were backfilled with an internal solution (in mM; 2.5 EGTA, 95 NMGCl, 5 NaCl, 10 CsCl, 2 MgCl_2_, 0.1 CaCl_2_, 10 HEPES, 5 ATP-bis(tris), and 5 glutathione). Data were considered for analyses if the seal resistance was greater than 2 GΩ, and access resistance was less than 7 MΩ.

After achieving a gigaohm seal, gentle suction was applied to form a whole-cell configuration. A holding potential of -110 mV was applied to all protocols to prime Na^+^ currents for activation. Activation was analyzed with depolarizing voltage steps from -70 to 60 mV, using a ΔV of 2.5 mV. Inactivation was studied using a standard protocol and range of pre-pulse from -110 to 60 mV, using a ΔV of 5 mV. All protocols employed p/n (n = 7) leak subtraction at the holding potential.

### Confocal Ca^2+^ imaging and measurement of SGNs

Isolated SGNs were loaded with the Ca^2+^ indicator Fluo 4-AM, following the manufacturer’s protocol (ThermoFisher Scientific, Waltham, MA) to monitor intracellular Ca^2+^ under a confocal microscope (Zeiss LSM 700). A line-scan was performed to measure the relative Ca^2+^ concentration across the SGN. Time-lapse recordings were performed to measure the dynamic changes of Ca^2+^ concentration in the selected region. Confocal line-scan imaging was performed by using a confocal microscope equipped with an argon laser (488 nm) and a 40X oil immersion objective lens (N.A., 1.3). Line-scan images were acquired at sampling rates of 0.7 ms per line and 0.07 μm per pixel, with radial and axial resolutions of 0.4 and 1.0 μm, respectively. Ca^2+^ transients were expressed as the normalized local fluorescence (F/F_o_), where F_o_ refers to the baseline fluorescence.

For cellular Ca^2+^ concentration measurements, we use the IonOptix Ca^2+^ measuring system (IonOptix LLC, Westwood, MA). SGNs were first loaded with Fura-2 (Thermo Fisher). After baseline recordings, the SGNs were stimulated by a continuous 10 V bipolar square pulse with 2 ms width at a frequency of 4 Hz. Cells were continuously perfused during the bath recordings solution. The 340/380 excitation ratio was recorded to determine the intracellular Ca^2+^ concentration changes.

### Statistical analyses

Where appropriate, pooled data are presented as means ± SD. Significant differences between groups were tested using t-test and ANOVA, where applicable. The null hypothesis was rejected when the two-tailed *p*-value < 0.05 is indicated with *, < 0.01 with **, < with ***. The number of mice and neurons are reported as *n*.
